# Machine learning based screening of potential paper mill publications in cancer research: methodological and cross sectional study

**DOI:** 10.1136/bmj-2025-087581

**Published:** 2026-01-29

**Authors:** Baptiste Scancar, Jennifer A Byrne, David Causeur, Adrian G Barnett

**Affiliations:** 1 IRMAR UMR 6625 CNRS, L’Institut Agro, Rennes, France; 2 NSW Health Statewide Biobank, NSW Health Pathology, Camperdown, NSW, Australia; 3 School of Medical Sciences, Faculty of Medicine and Health, University of Sydney, Camperdown, NSW, Australia; 4 School of Public Health and Social Work, Queensland University of Technology, Kelvin Grove, QLD 4059, Australia

## Abstract

**Objectives:**

To train and validate a machine learning model to distinguish paper mill publications from genuine cancer research articles, and to screen the cancer research literature to assess the prevalence of papers that have textual similarities to paper mill papers.

**Design:**

Methodological and cross sectional study applying a BERT (bidirectional encoder representations from transformers) based, text classification model to article titles and abstracts.

**Setting:**

Retracted paper mill publications listed in the Retraction Watch database were used for model training. The cancer research corpus was screened by the model using the PubMed database restricted to original cancer research articles published between 1999 and 2024.

**Population:**

The model was trained on 2202 retracted paper mill papers and validated on independent data collected by image integrity experts. 2.6 million cancer research papers were screened.

**Main outcome measures:**

Classification performance of the model. Prevalence of papers flagged as similar to retracted paper mill publications with 95% confidence intervals and their distribution over time, by country, publisher, cancer type, research area, and within high impact journals (top 10%).

**Results:**

The model achieved an accuracy of 0.91. When applied to the cancer research literature, it flagged 261 245 of 2 647 471 papers (9.87%, 95% confidence interval 9.83 to 9.90) and revealed a large increase in flagged papers from 1999 to 2024, both across the entire corpus and in the top 10% of journals by impact factor. More than 170 000 papers affiliated with Chinese institutions were flagged, accounting for 36% of Chinese cancer research articles. Most publishers had published substantial numbers of flagged papers. Flagged papers were overrepresented in fundamental research and in gastric, bone, and liver cancer.

**Conclusions:**

Paper mills are a large and growing problem in the cancer literature and are not restricted to low impact journals. Collective awareness and action will be crucial to address the problem of paper mill publications.

## Introduction

Research paper mills are “contract-cheating organisations which provide undeclared services to support research manuscripts and publications.”^1^ Research paper mills fabricate and submit manuscripts for their customers. The first research paper mill activity was reported in the 2010s,^2^ and has since increased in volume and sophistication. More than 400 000 papers suspected to have originated from paper mills have been published in the past 20 years,^3^ with the paper mills earning tens of millions of dollars annually.^4^ This issue gained visibility when Wiley—after acquiring Hindawi—retracted nearly 11 000 suspected paper mill papers and shut down 19 journals over two years.^5^

Research paper mills maximise their earnings by quickly producing industrial quantities of low quality research papers.^6^ ^7^ To produce manuscripts at scale, fabrication has likely relied on templates with premade sentences where domain specific terms vary.^8^ Suspect papers can include incorrect reagents,^9^ fabricated data and experiments,^2^ ^10^ and photoshopped or reused figures.^11^ Paper mill papers are often generic, poorly written, lack coherence between sections,^8^ ^12^ and may offer superficial research justifications.^7^ Paper mills sell manuscripts to researchers eager to increase their number of publications,^10^ creating author groups who have never worked together or made any intellectual input.^13^ Paper mills often cite their own productions,^14^ possibly as part of their service to clients who pay for citations.^15^ Paper mills may even bribe editors and manipulate peer review to facilitate publication, as shown by online discussions between researchers and likely paper mill contacts.^4^

Paper mill manuscripts can be simultaneously submitted to several journals until acceptance, wasting the time of editors and reviewers.^2^ ^11^ ^16^ The reported proportion of paper mill submissions to journals ranges from 2% to 46%.^2^ Paper mills likely target journals where fabricated manuscripts have already been accepted,^2^ increasing their chances of success. They may also focus on high impact journals because the prices they charge—according to Abalkina’s investigations of a paper mill^16^—can be directly linked to the journal’s impact factor.^17^

The overall prevalence of paper mill papers in biology and medicine is estimated to be 3%,^3^ but cancer research and particularly molecular oncology could be more affected.^1^ This can be explained by high publication pressure,^7^ a specialised field with simple-to-fake data and techniques,^1^ and limited peer review capacity^6^—making fake papers easier to produce and harder to detect.

The rise of artificial intelligence (AI) could exacerbate the paper mill problem through automated image and text generation, making detection more challenging.^18^ Some publishers are using screening tools to detect manuscripts from paper mills.^19^ ^20^ Integrity sleuths have also developed detection methods, such as identifying awkward rewording of scientific terms, known as “tortured phrases,”^21^ or nucleotide sequence reagent verification.^9^ Paper mill manuscripts may have missing or unusual acknowledgments, funding, or ethics statements.^11^

Previous publications have documented recurrent formatting templates among suspected paper mill papers, including identical layouts and nearly identical textual formulations in figure legends.^8^ ^9^ ^13^ ^22^ ^23^ Bless and colleagues^24^ reported “distinct phrase patterns and higher word repetition” and “lower lexical diversity” in full texts and abstracts of retracted papers. Also, Bless and colleagues^24^ and other studies^25^ ^26^ have shown that machine learning methods can predict retractions and paper mill products from text using data from Retraction Watch—a non-profit group that records paper retractions^27^—in a cross disciplinary context rather than within a specific scientific domain.

The performance of such an approach has never been tested in cancer research, and we believe it has the potential to systematically screen papers to assess the prevalence of papers sharing textual similarities with paper mill publications. We hypothesise that paper mills use text templates, which likely extend to titles and abstracts. We also believe that these templates are domain and publication type specific and could constitute strong signals to machine learning models. Therefore, we aim to use cancer research titles and abstracts from retracted paper mill papers as input to a BERT based (bidirectional encoder representations from transformers)^28^ machine learning pipeline for a text classification task. BERT learns from examples to recognise patterns in text, enabling it to identify when new papers share similarities with retracted paper mill papers. Using only titles and abstracts enables easy access to training data and offers scalability for producing broad estimates.

The first objective of this study was to train and evaluate our model’s ability to reliably classify titles and abstracts from retracted papers attributed to suspected paper mill activity and genuine cancer research papers. The second objective was to use our model to screen millions of cancer research papers to assess the prevalence of flagged papers over time, across countries, publishers, and cancer research subdomains, and to examine how they have evolved in high impact factor journals (defined as decile 1 or D1—the top 10% of journals in this study). Throughout, we use flagged papers to refer to articles whose titles and abstracts are textually similar to retracted papers tagged as “paper mill” in Retraction Watch. Flagging is a statistical screen, not an attribution of misconduct.

This research aims not only to assess the potential of machine learning for detecting cancer research papers that share textual features with paper mill manuscripts, but also to raise awareness of potentially fabricated papers among stakeholders in cancer research. We believe that such research could support editorial triage, help protect genuine researchers from citing or using fake research, inform funding and institutional policies on research integrity, and provide insights into the scale and dynamics of paper mill activity within cancer research.

## Methods

### Cancer research corpus

#### PubMed database preprocessing

To create a comprehensive cancer research dataset, the entire biomedical research corpus from the PubMed database (https://pubmed.ncbi.nlm.nih.gov/) was downloaded in March 2025. The following data were extracted from each of more than 38 million papers: PubMed Identifier (PMID), title, abstract, original language, journal name, journal ISSN (International Standard Serial Number), publication date, first author’s affiliation, publication type, and MeSH terms (medical subject headings). The data were preprocessed following the method used by González-Márquez and colleagues.^29^ We excluded abstracts that were non-English, empty, truncated, and unpunctuated to avoid these features influencing the language model. The text was transformed into standardised tokens (units of text such as words or punctuation marks) for analysis. Abstracts of less than 250 tokens and more than 4000 tokens were removed because these were generally non-standard abstracts and were also rare (<1%). After this initial filtering, the research dataset contained 24.8 million papers published between 1975 and 2025.

Next, the dataset was filtered to retain only papers published within the target timeframe (1999-2024): papers before 1999 or after 2024 were excluded, and duplicates were removed, reducing the dataset to 20.2 million papers. We only included papers classified as “journal article” and excluded other publication types, such as literature reviews and clinical trials. These types may also be targeted by paper mills, but would require separate models because we expect paper mills to use manuscript type specific templates. All retraction notices, corrections, and expressions of concern were also removed. After this second filtering step, 17.4 million papers remained.

#### Cancer research filtering

The cancer research corpus was derived from the remaining papers using a two level keyword filtering strategy. Box 1 shows the keywords searched for in titles and abstracts of the 17.4 million papers. These keywords were adapted from MeSH terms and National Cancer Institute^30^ terminology. The keyword matching was designed to be specific to cancer while also retaining the broadest coverage of cancer research papers. We acknowledge that some non-cancer related papers may be included and that not all cancer specific terms have been used.

Box 1 **Cancer related keywords**

astrocytoma, carcinoembryonic antigen, carcinoid, carcinogen, carcinogenesis, carcinoma, cancer, checkpoint inhibitor, chemotherapy, chordoma, ependymoma, glioblastoma, glioma, leukaemia, leukemia, lymphoma, macroglobulinaemia, macroglobulinemia, medulloblastoma, melanoma, mesothelioma, metastasis, metastatic, myelodysplastic syndrome, myeloma, myeloproliferative neoplasm, neuroblastoma, nsclc, oncogene, oncogenesis, oncology, pheochromocytoma, radiation therapy, radiotherapy, retinoblastoma, sarcoma, seminoma, tumor, and tumour.

Substring matching ensured that terms such as “osteosarcoma” were captured under broader categories like “sarcoma.” Papers matching multiple keywords were included only once to avoid duplicates. UK and US spellings of each cancer related term were used. All levels of the search were combined to produce a final cancer research dataset of 2 647 471 papers published across 11 632 journals. Supplementary file 1 provides a flowchart detailing the filtering strategy from the initial PubMed database to the final cancer research corpus.

Data extracted for visualisation purposes are the first author's country of affiliation, the publisher, the type of cancer investigated, the main cancer research areas, and the SCImago journal impact factor.^31^ The type of cancer investigated and the main cancer research areas were inferred from the titles, abstracts, and MeSH terms of the papers using AI based labelling. Supplementary file 2 describes all extraction methods. A detailed breakdown of these variables describing the cancer research corpus is provided in supplementary file 3.

### Paper mill datasets

We developed our model using two sources of paper mill papers: papers tagged as originating from paper mills in the Retraction Watch database^27^; online lists compiled by image integrity experts—also called integrity sleuths—where evidence of image manipulation was found. A compilation of paper mill papers is available online in the “Spreadsheet of spreadsheet” thanks to anonymous PubPeer contributors.^32^ PubPeer^33^ is a website (https://pubpeer.com/) that allows users to leave comments after publication about potential research integrity issues or other concerns. The website has been used in research on integrity^34^ and has played an important role in high profile retractions.^35^

The Retraction Watch dataset was used for model development (training, optimisation, and internal validation), whereas the experts’ dataset was reserved for external validation of model performance. From the 64 457 total retractions recorded in the Retraction Watch database as of June 2025, we removed papers that were not in the PubMed database, and those that were not retractions (eg, expressions of concern, corrections, or reinstatements). The “Paper Mill” tag in the retraction reason field was used to identify 5657 retracted publications. Only those whose PMIDs matched entries in our cancer research corpus were retained, reducing the number to 2270 retracted papers. We excluded papers for which the original text had been replaced by the retraction notice, reducing the number of retracted paper mill papers to 2202.

To validate the model’s ability using new data, we used 3094 suspected paper mill papers from the integrity experts’ dataset for external model validation, excluding those that overlapped with the Retraction Watch set. These papers were also extracted from the cancer research corpus.

Supplementary file 4 presents visualisations of data used at the training stage from the Retraction Watch and the integrity experts’ datasets. These data show the distribution of publishers, countries of the first authors’ institutions, cancer types, and research areas among paper mill papers, as well as the single word, two word, and three word combinations most prevalent in the titles of paper mill papers.

### Model selection and training

#### Controls selection

Our training dataset was chosen to be balanced, with 50% paper mill papers and 50% presumed genuine papers. Controls were selected from the cancer research corpus with the aim of including as few paper mill papers as possible to minimise bias and enhance training performance. Given the difficulty of assessing the genuine status of cancer research papers in large samples, control papers were selected from high impact factor journals (decile 1) and countries under-represented in the Retraction Watch database (using the country of the first author’s institution). Throughout this paper, controls should be regarded as proxies for quality research, not verified genuine papers.

To avoid a potential bias in the English diction used by Chinese authors, we included 101 papers (5%) in the control set authored by researchers from Chinese institutions that were published in four high impact journals: *Cell*, *Cancer Cell*, *Molecular Cell*, and *The EMBO Journal*. We also included 600 of the most cited Taiwanese cancer research papers (28%) listed in OpenAlex^36^ because Taiwan is a predominantly Mandarin speaking country, but is less represented in the Retraction Watch database (with only one recorded retraction owing to paper mill involvement in the cancer research corpus). Another 33% of the control papers were randomly selected from Swedish, Finnish, and Norwegian institutions in cancer research because these countries have no recorded instances of paper mill retractions in the Retraction Watch database. The remaining 33% consisted of a random selection of papers published in *Cell*, *Cancer Cell*, *Molecular Cell*, and *The EMBO Journal* from countries other than China, Taiwan, Norway, Sweden, and Finland.

For the external validation dataset, papers from the integrity experts’ dataset were combined with a similar number of control papers randomly sampled from authors from Swedish, Finnish, and Norwegian institutions, and other papers published in the four high impact factor journals mentioned above. None of the controls or paper mill papers used in the training set overlap with those in the external validation set.

All papers in the control sets were manually verified as free of research integrity concerns on PubPeer (June 2025). A paper was removed if any problem was reported. We assumed that all papers in the paper mill datasets are indeed paper mill products, and that the controls are legitimate scientific papers. However, we acknowledge that the ground truth is unknown and that, despite our efforts, some papers may have been mislabelled.

#### Model selection and training

We chose to use only titles and abstracts to train the model because these data were often available (full texts are frequently behind paywalls). Each paper’s title and abstract were combined. We framed the detection of paper mill papers as a binary text classification problem—authentic or fraudulent—to provide supervision signals to the model.

The 4404 labelled papers from the Retraction Watch dataset and its controls (2202 paper mill papers and 2202 controls) were split into 70% for training (n=3082), 17.5% for optimisation (n=771), and 12.5% for internal validation (n=551). The model was trained and tuned using the training and optimisation sets. Splitting was performed at the paper level and stratified by class label to preserve the 1:1 ratio of paper mill and control papers across all subsets.

Model performance (accuracy, sensitivity, and specificity) was first evaluated on the internal validation set—which was never seen during training or optimisation—and then on an external validation set composed of papers from research integrity experts (3094 paper mill papers and 3100 controls). The latter dataset was entirely independent from Retraction Watch and used to assess the model’s generalisation to new data. Table 1 provides a summary of all datasets and their respective roles. Each metric was derived from the confusion matrix using a probability threshold to prioritise specificity and minimise false positives. Reported values correspond to a single final model obtained after hyperparameter optimisation over 150 trials. Confidence intervals were not computed because performance was estimated on fixed validation sets. Supplementary file 2 gives further details.

**Table 1:** Summary of the five datasets and their use in the experiment design

Source	Purpose	Sample size
Retraction Watch	Model training	1541 cases and 1541 controls
Retraction Watch	Model optimisation	385 cases and 386 controls
Retraction Watch	Internal validation	276 cases and 275 controls
Integrity experts	External validation	3094 cases and 3100 controls
Cell lines and nucleotide sequences	Unlabelled testing	873 papers with likely high paper mill representation

We selected BERT to analyse the text for its strong performance and relatively low computational cost because of its moderate number of parameters.^28^ The model was initialised from the public BERT base uncased checkpoint and fine tuned on our dataset with a new classification head. The model was initially compared with RoBERTa, BioBERT, PubMedBERT, Longformer, and Clinical Longformer^37^ ^38^ ^39^ ^40^ ^41^ to assess the potential of biomedical specific pretraining and extended input capacity. Supplementary file 5 gives details of comparative model testing.

All journal specific formatting was removed from the abstracts. Because BERT truncates inputs beyond its 512 token limit, each title and abstract were split into individual sentences to prevent information loss. Each sentence was labelled (paper mill or not) and fed into BERT during the training phase. During inference, predictions were made at the sentence level and final classification probabilities were obtained by averaging the positive class probabilities across the title and abstract. Supplementary file 2 describes the optimisation method. A logistic regression model was also tested on BERT’s output probabilities to incorporate sentence ordering; however, it did not outperform the simple averaging approach and so was not used (supplementary file 2).

#### Additional validation

To further evaluate the model, we checked whether 873 problematic cancer research papers reported in three previous studies where paper mill involvement was suspected^42^ ^43^ ^44^ were flagged by the model (table 1). These papers were not necessarily from paper mills because similar errors can sometimes occur in genuine research. This analysis was not intended to measure model performance directly, but rather to assess the model’s ability to flag papers that had already been reported as suspicious. Although we did not expect all these papers to be flagged, we did anticipate that most would be.

None of these papers were included in the training or validation sets. All papers overlapping with paper mill papers or controls from the training, optimisation, or validation sets were removed during data extraction (n=29). No content re-evaluation was performed, and integrity related information was not disclosed to the model. These papers, involving misidentified or non-verifiable nucleotide sequences (primers and other oligonucleotides used for amplification, detection, or gene knockdown), or both, or cell lines, were retrieved from three sources: 193 cancer related papers identified by Oste and colleagues,^42^ 113 cancer related papers in high impact factor journals—*Molecular Cancer* and *Oncogene*—reported by Pathmendra and colleagues,^43^ and 567 cancer related papers listed by Park and colleagues.^44^

### Screening the cancer literature

Each of the 2.6 million papers of the cancer research corpus published between 1999 and 2024 was screened with our fine tuned version of BERT trained to identify papers with textual features associated with retracted paper mill papers. We refer to papers that were classified as resembling retracted paper mill papers as “flagged papers.” None of the papers seen by the model during training were excluded from the cancer research corpus (n=3853). These papers were considered part of the corpus and represented a small percentage of flagged papers and so not large enough to influence the overall conclusions.

The 95% confidence interval for the proportion of flagged papers was estimated through bootstrapping (1000 resamples with replacement). All figures are presented as percentages of the total number of papers to illustrate the proportion of non-flagged papers compared with flagged papers. Data were visualised using the ggplot2 R library.^45^ Supplementary file 6 gives a diagram of the study design.

### Patient and public involvement

There was no involvement in this research of patients or the public because publication policy is indirectly related to patient health and the key stakeholders are publishers and other researchers.

## Results

### Model performance and errors

#### Training data

Retraction Watch covered papers from 2007 to 2024, with a peak in 2022, while the integrity experts’ data included papers from 2010 to 2024, with a peak between 2019 and 2020 (supplementary figure S4). In the Retraction Watch dataset, the most frequent publisher is John Wiley and Sons (via Hindawi), followed by Spandidos Publications, and Informa (supplementary table S4.2). In the experts’ dataset, Springer Nature has the most suspected paper mill papers, followed by John Wiley and Sons, and Spandidos Publications (supplementary table S4.4). Papers from Chinese institutions are highly represented in both datasets (supplementary tables S4.2 and S4.4). Common topics in paper titles for both datasets include microRNAs, long non-coding RNAs, and lung cancer related terms (supplementary tables S4.1 and S4.3). Many papers cover topics related to gene and preclinical research. The most frequent cancer types are lung, liver, colorectal, gastric, and brain cancer (supplementary tables S4.2 and S4.4). The most prevalent research fields are cancer biology and cancer therapy, followed by cancer diagnosis (supplementary tables S4.2 and S4.4).

#### Performance

Table 2 summarises model performance metrics for both datasets. The classification model achieved an accuracy of 0.91 in detecting paper mill papers in the Retraction Watch dataset (502 of 551 papers were correctly classified as paper mill associated or genuine), with a sensitivity of 0.87 (239 of 276 paper mill papers correctly identified) and a specificity of 0.96 (263 of 275 genuine papers correctly identified). The titles and abstracts of three retracted paper mill papers, all correctly predicted with high probability, are presented as examples in supplementary file 7. In validating on unseen data for the integrity experts’ dataset, the model had a similar classification accuracy of 0.93 (5771 of 6194) with a sensitivity of 0.87 (2698 of 3094) and a specificity of 0.99 (3073 of 3100).

**Table 2:** Model performance over internal and external validation sets

Set	Accuracy	Sensitivity	Specificity
Internal validation	91% (502/551)	87% (239/276)	96% (263/275)
External validation	93% (5771/6194)	87% (2698/3094)	99% (3073/3100)

In further validation sets, the model flagged 67% (130 of 193) of problematic papers found by Oste and colleagues,^42^ 75% (425 of 567) of problematic papers found by Park and colleagues,^44^ and 66% (75 of 113) of problematic papers found by Pathmendra and colleagues.^43^ About 72% of the combined problematic papers were flagged by the model.

#### Misclassifications

We combined the misclassifications from the internal and external validation sets because their characteristics were similar (supplementary file 4). Pooling these data increased the sample size and therefore improved the robustness of analyses. False positives—control papers incorrectly predicted to resemble paper mill papers—were rare, with only 39 out of 3375 across the Retraction Watch and integrity experts’ datasets. This small number of false positives did not allow for the identification of generalisable patterns. In contrast, 433 out of 3370 paper mill papers were false negatives—paper mill papers incorrectly predicted as authentic.

Table 3 summarises the main characteristics of false negative predictions by the model and an extended analysis is given in supplementary file 8. False negatives are disproportionately associated with first authors affiliated in China as they account for 90% of false negatives (compared with 47% in the overall validation dataset). Papers published in 2014 (+9%) and 2015 (+8%) were more often misclassified as false negatives by the model. Gastric cancer (+4%), liver (+4%), colorectal (+4%), and lung cancer (+4%), as well as publishers such as Rapamycin Press LLC (+4%) and John Wiley and Sons (+2%) are slightly overrepresented among the false negatives. Epidemiology papers are slightly overrepresented among false negatives, while text analyses do not show overrepresented topics other than general cancer words in the false negatives (supplementary file 8).

**Table 3:** Characteristics of false negatives (n=433) by publication year, publisher, first author’s country, and cancer type

Variables and most overrepresented categories	False negative (%)	Overall (%)	Difference (%)	χ^2^ P value
**Year**				
2014	12	3	9	<0.001
2015	13	5	8	
**Publisher**				
Rapamycin Press LLC	7	3	4	<0.001
John Wiley and Sons	14	12	2	
**Country**				
China	90	47	43	<0.001
**Cancer type**				
Gastric cancer	8	4	4	<0.001
Liver cancer	10	6	4	
Colorectal cancer	12	8	4	
Lung cancer	12	8	4	

For each variable, the 10 most frequent categories were retained. A single Pearson’s χ^2^ test of independence was carried out for each variable. Categories listed that were significantly overrepresented for each variable are defined by standardised residuals >2 (this is not tested and only descriptive). Percentages are shown within the false negatives and overall pooled validation dataset (n=6745). The difference is the false negative minus overall. Data are percentages only; absolute counts not shown.

### Flagged papers in cancer literature

After applying our model to the cancer research corpus from 1999 to 2024, 261 245 papers were flagged as including textual characteristics of retracted paper mill papers. This number represents 9.87% (95% confidence interval 9.83 to 9.90) of all original cancer research papers.

#### Trends in flagged papers

**Fig 1:**
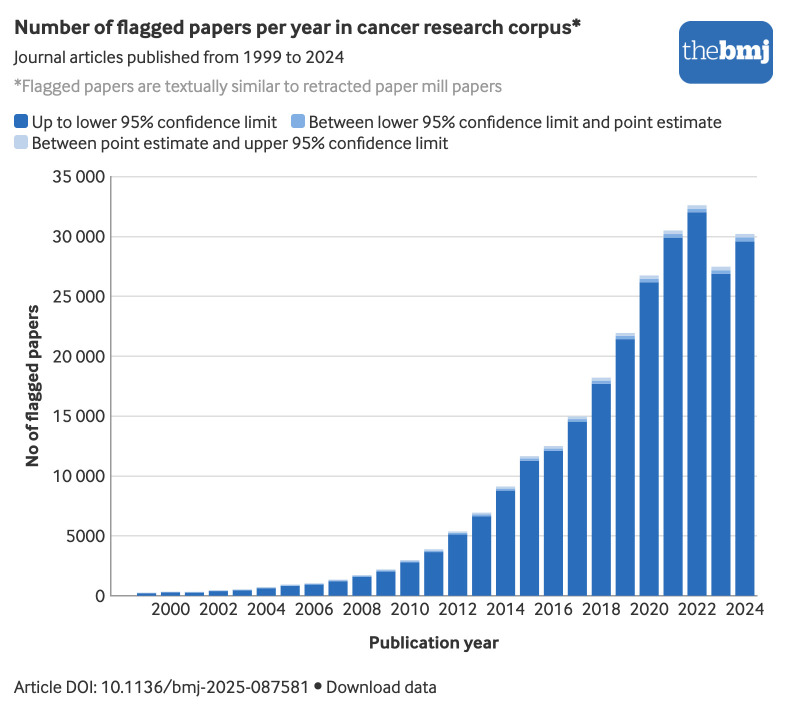
Number of papers each year in cancer research corpus flagged because their titles and abstracts were similar to those of retracted paper mill papers. 95% confidence intervals estimated using bootstrap resampling. An interactive version of this graphic and downloadable data are available at  https://public.flourish.studio/visualisation/27240897/

The number of flagged papers showed a clear and rapid increase between 1999 and 2022 (fig 1), peaking in 2022 before showing a slight decline in 2023 and 2024. The number of flagged papers each year followed an exponential trend from 1999 to 2022 (R^2^ for exponential fit=0.92). Although the percentages of flagged papers remained around 1% in the early 2000s, they progressively rose to exceed 15% (26 457/171 656) of the annual cancer research output by the early 2020s.

#### Countries of flagged papers

**Fig 2:**
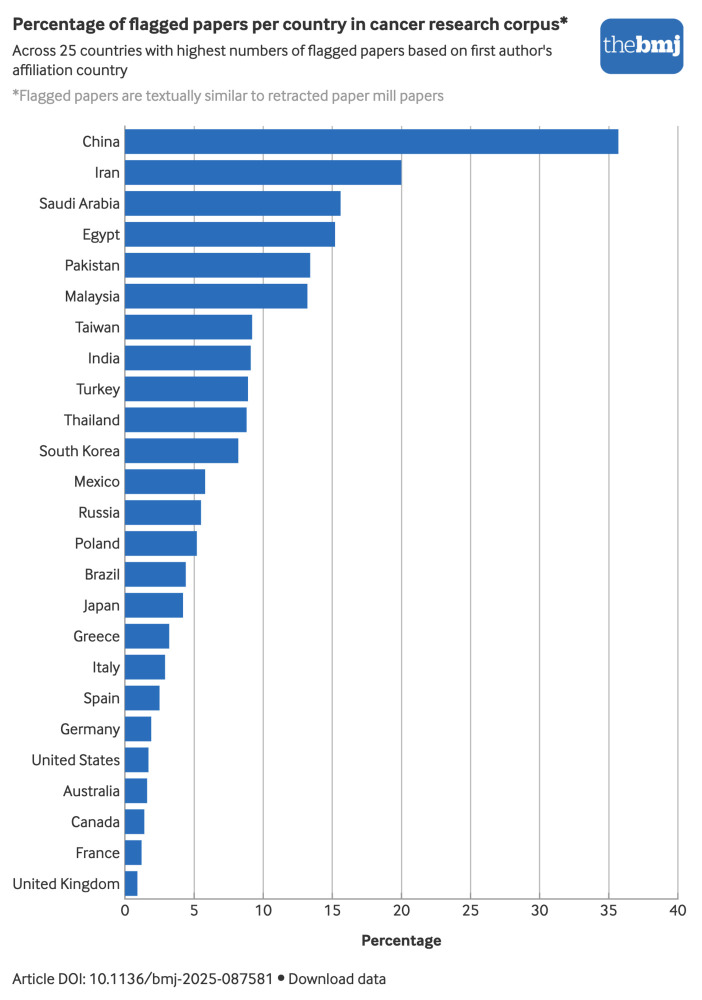
Percentage of papers in cancer research corpus flagged because their titles and abstracts were similar to those of retracted paper mill papers across 25 countries with highest numbers of flagged papers based on first author's affiliation country. An interactive version of this graphic and downloadable data are available at https://public.flourish.studio/visualisation/27226146/

The percentages of flagged papers for each country show that papers from China were most frequently flagged, representing 36% of cancer papers with 177 907 of 497 672 flagged papers (fig 2), followed by Iran with 20% of flagged papers, accounting for 6801 of 33 935 papers. Papers from four other countries were also frequently flagged: Saudi Arabia (16%, 1607/10 308), Egypt (15%, 2229/14 618), Pakistan (13%, 883/6578), and Malaysia (13%, 870/6615). The United States was the second country in terms of number of flagged papers, with 10 511 flagged papers, representing 2% of US cancer research papers.

#### Publishers and journals of flagged papers

**Fig 3:**
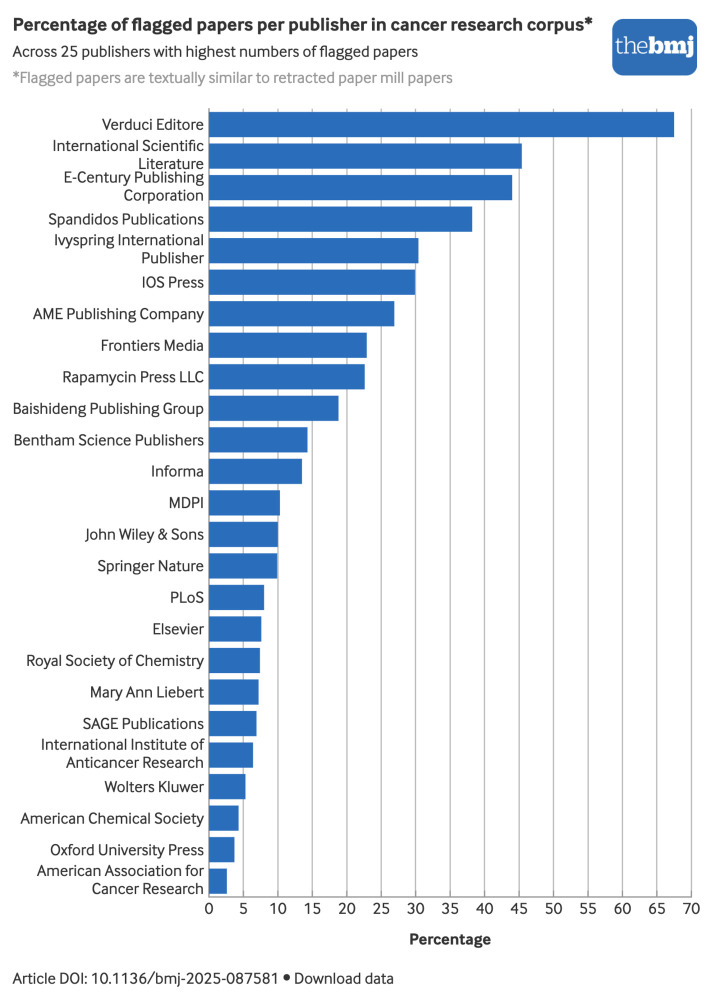
Percentage of papers in cancer research corpus flagged because their titles and abstracts were similar to those of retracted paper mill papers for 25 publishers with highest numbers of flagged papers. An interactive version of this graphic and downloadable data are available at https://public.flourish.studio/visualisation/27226940/

The publisher Verduci Editore had the highest percentage of flagged papers with about 67% (2834/4199) in its cancer research journal—*European Review for Medical and Pharmacological Sciences* (fig 3). The second publisher in terms of percentage was International Scientific Literature, with about 45% (1261/2776) of papers flagged in one journal—*Medical Science Monitor (MSM)*. The next four publishers were E-Century Publishing Corporation (44%, 4620/10 510), Spandidos Publications (38%, 19 043/49 796), Ivyspring International Publisher (30%, 3065/10 069), and IOS Press (30%, 3079/10 302).

The largest publishers—such as Springer Nature, Elsevier, and John Wiley and Sons—have a relatively low percentage of flagged papers (around 10%), but account for the highest absolute numbers of flagged papers across more than 500 journals, with flagged paper numbers of 40 293 (Springer Nature), 39 753 (Elsevier), and 28 330 (John Wiley and Sons).

#### Cancer types of flagged papers

**Fig 4:**
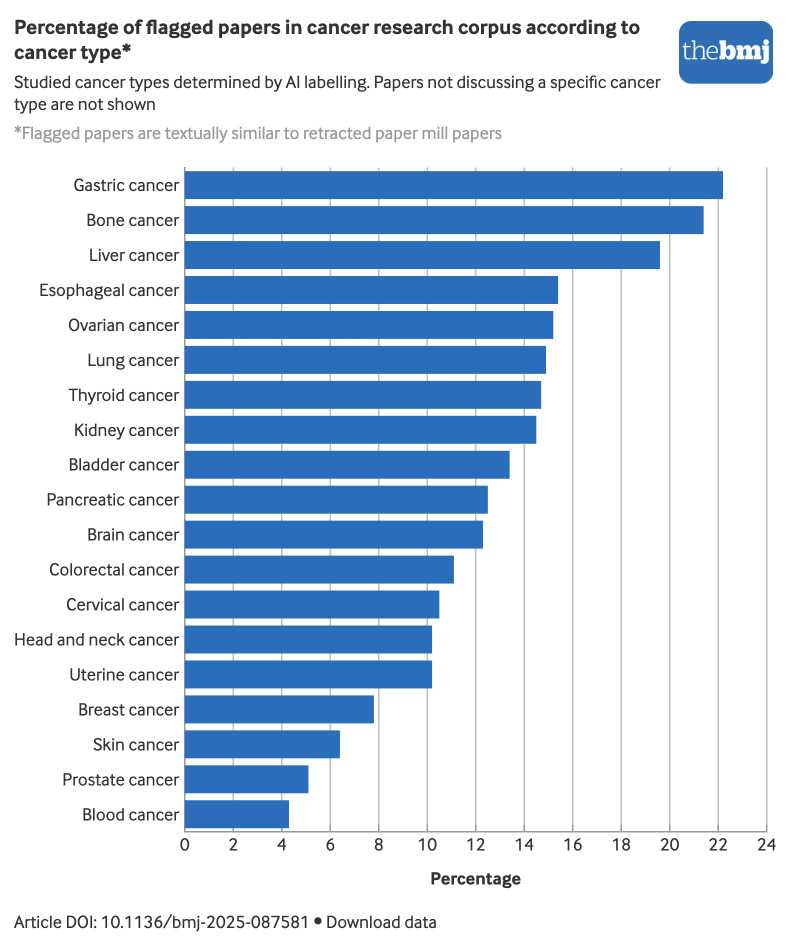
Percentage of cancer research papers according to cancer type flagged because their titles and abstracts were similar to those of retracted paper mill papers. An interactive version of this graphic and downloadable data are available at https://public.flourish.studio/visualisation/27227226/

Among all cancer types, gastric cancer papers show the highest percentage of flagged papers, with 22% (18 398/82 690) of these papers flagged (fig 4). Bone cancers, such as osteosarcoma, follow with 21% (8458/39 433) of papers flagged, followed by liver cancer at 20% (26 730/136 719). Most cancer types fall within a range of 10-15% of flagged papers. Breast, skin, prostate, and blood cancers show the lowest percentages of flagged papers. In terms of absolute numbers, lung (28 435) and liver (26 730) cancer account for the highest numbers of flagged papers.

#### Cancer research area of flagged papers

**Fig 5:**
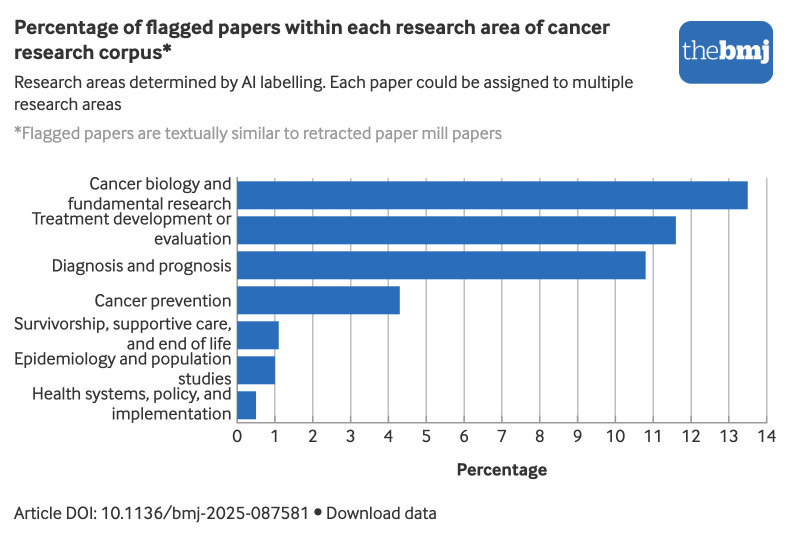
Percentage of cancer research papers within each research area flagged because their titles and abstracts were similar to those of retracted paper mill papers. Because categories were assigned using a multi-labelling tool, each paper may appear in several categories and sum of all categories does not equal total number of flagged papers. Percentages represent proportion of papers flagged as similar to retracted paper mill papers within each research area, calculated as (flagged papers in area/total papers in area)×100. An interactive version of this graphic and downloadable data are available at https://public.flourish.studio/visualisation/27227359/

Flagged papers are largely concentrated in cancer biology and fundamental research, as well as in treatment development or evaluation and in diagnosis and prognosis, with percentages exceeding 10% (fig 5). By contrast, areas such as survivorship, supportive care, and end of life; epidemiology and population studies; and health systems, policy, and implementation had lower percentages of flagged papers (<2%).

#### Flagged cancer research papers in high impact factor journals

**Fig 6:**
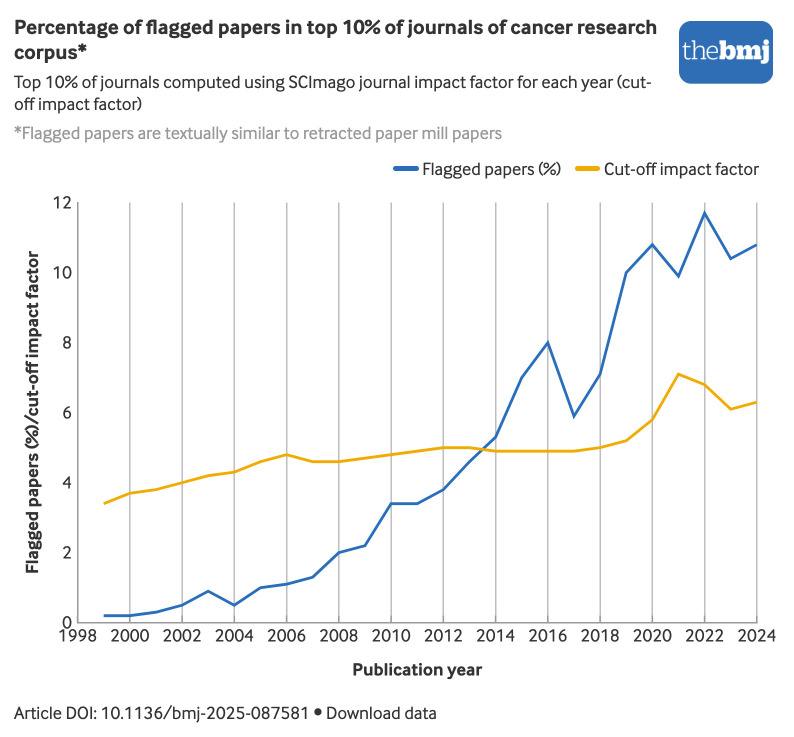
Percentage of cancer research papers flagged because their titles and abstracts were similar to those of retracted paper mill papers in top 10% of journals by impact factor according to publication year. An interactive version of this graphic and downloadable data are available at https://public.flourish.studio/visualisation/27227402/

The percentage of flagged papers in the top 10% of journals from the cancer research corpus by impact factor (decile 1) shows a clear increase over time (fig 6). While the percentage remained low in the early 2000s, a sustained increase occurred in the following years, reaching over 10% in 2022 (3383/28 991). The minimum impact factor required to be among the top 10% of journals for each year (cutoff impact factor) also increased from 3 in 1999 to 7 in 2021.

## Discussion

### Principal findings

We have fine tuned a BERT machine learning model that achieves good accuracy in flagging suspected paper mill papers, using combined titles and abstracts from Retraction Watch and image integrity experts’ datasets. The model also flagged many papers previously identified as containing incorrect nucleotide sequences or cell lines,^42^ ^43^ ^44^ despite not having access to this information, showing its ability to detect problematic papers without circular validation.

The model’s performance on Retraction Watch and integrity experts’ data supports the hypothesis that paper mills can rely on textual templates to create the titles and abstracts of cancer research papers. Although some of the text signals might be because of geographical writing styles, specific terminology, or area specific research topics, the analysis of misclassified papers showed little systematic bias with a low number of false positives. Concerns about our model only identifying the writing style of Chinese authors are somewhat ameliorated by the high number of false negatives for authors from China, whereas we would be concerned about undue geographical discrimination if any country was overrepresented in the false positives.

The model flagged almost 10% of cancer papers, a higher proportion than the previous 3% estimate of paper mill paper prevalence in biomedical research,^3^ although those estimates were derived from different datasets, time periods, and detection criteria. The positive predictive value depends on the true prevalence of paper mill papers, which remains unknown but is likely higher in cancer research.^1^ Assuming a 10% true prevalence and the observed model performance (sensitivity and specificity), the positive predictive value would be approximately 0.7, meaning that around 30% of flagged papers would represent false positives. This reinforces the need to not solely rely on the model prediction to conclude that a manuscript is from a paper mill.

The exponential rise of flagged papers in figure 1 flattens after 2022. Three potential hypotheses can explain this phenomenon: the result of the publishers and research community fighting back against paper mills; a shift to new templates by paper mills after the rise of AI; and the known delays in PubMed listing all papers for the most recent years. The relatively low number of flagged papers before 2010 may reflect the distribution of the training data, which primarily includes retracted paper mill papers published between 2013 and 2023, rather than indicating a near complete absence of such features during these years. This finding could also reflect the use of proxy labels rather than verified ground truth. Some unlabelled older papers may be paper mill products, inflating apparent time trends and contributing to temporal drift.

The higher proportion of flagged papers in gastric and liver cancer research may partly be explained by the high prevalence of these cancers in China.^46^ However, their marked overrepresentation among misidentified cell lines—25% and 15% of all such lines, respectively^47^—is striking. Given that some misidentified cell lines, such as BGC-823 and BEL-7402, appear almost exclusively in publications from Chinese institutions,^47^ this pattern may also reflect vulnerabilities exploited by paper mills when popular research topics are targeted. Furthermore, this pattern could result from inertia because early templates were reused and adapted repeatedly in these domains.

The rise in the percentage of flagged papers in high impact journals suggests that paper mill papers are not just a problem for low impact journals. The concurrent increase in impact factors and the spread of flagged papers suggest that both phenomena may stem from the pressures of the publish-or-perish culture.^48^ The increase in flagged papers in high impact factor journals highlights an important limitation of using impact factors as proxies for research quality.^43^

### Strengths and limitations of the study

The consistent model performance across different validation sets confirms its ability to reliably identify textual features characteristic of paper mill papers and strengthens the hypothesis that these papers share common title and abstract templates. Several indirect indicators support the validity of the model’s predictions: the exponential trend over time in flagged papers coincides with the known development of paper mill papers.^2^ China being the leading country in terms of flagged papers is consistent with findings of paper mill paper origins.^9^ ^49^ ^50^ Publishers with a high percentage of flagged papers in this study have been found to publish problematic papers, and some have been flagged as “in conflict with academic rigour” by the Chinese government.^50^ ^51^ ^52^ Flagged papers were more common in fundamental research, which coincides with evidence from the literature.^50^ Finally, suspected paper mill papers have already been identified in high impact factor cancer journals.^43^

Our training set of paper mill papers has limitations. The tag “paper mill” in the Retraction Watch database reflects only Retraction Watch staff’s interpretation of the publisher’s retraction notice. No uniformity exists in the way publishers investigate fraudulent papers and there is no standard way of interpreting the retraction notices; therefore, the “paper mill” qualification likely reflects varied levels of evidence. The papers listed online by research integrity experts include evidence of image manipulation, which can occur within settings beyond paper mills. Additionally, experts’ methods and transparency may vary. Research on paper mills remains limited,^1^ which could mean that the currently identified paper mill papers represent only a fraction of their actual prevalence in the scientific literature. Consequently, the model will likely not detect textual features associated with paper mill papers as a whole, but rather those represented in the training set.

The overrepresentation of authors from Chinese institutions among retracted papers suspected of originating from paper mills introduces a potential bias. Despite efforts to balance by language in the control set, a residual risk remains that the model may learn to associate linguistic patterns of Chinese scientific writing with paper mill content, rather than identifying features specific to fraudulent manuscripts. However, the model misclassification analysis shows few false positives, and an overrepresentation of Chinese papers among false negatives—which does not indicate systematic over-flagging. Furthermore, the paper mills’ country of origin can differ from the authors’ countries and Abalkina showed that a Russian paper mill sold publications to “more than 800 scholars affiliated with more than 300 universities from at least 39 countries.”^16^ The misclassifications may instead reflect blind spots in the training data where certain textual features in the wider population of paper mill papers were underrepresented or absent in our training data.

Additional sources of bias may stem from the composition of the control set. Controls were not randomly sampled from the broader cancer research literature to avoid including undetected paper mill papers. As a result, only a limited number of verified high quality Chinese publications were included in the controls to minimise potential overlap with paper mill content. The assumption—supported by retraction data—that articles published in selected high impact journals or authored by Taiwanese, Swedish, Norwegian, and Finnish research teams can serve as proxies for high quality controls is open to criticism. Although this strategy may enhance contrast between genuine and fabricated texts, it may also limit the model’s ability to detect more nuanced cases of fabrication. We also acknowledge that only limited human verification was conducted to assess the validity of the ground truth labels, and that potential misclassifications may have affected the model’s quality. Future research could manually verify a larger and more diverse set of Chinese publications to improve label accuracy.

The non-explainability of deep learning models prevents us from directly identifying the features captured by BERT. Flagged papers can include actual paper mill features; other features of misconduct; original work copied by paper mills; original work drawing inspiration from paper mill papers; and mistakes by the model. This research does not aim to directly identify paper mill papers or to accuse anyone of fraud. It focuses on studying overall patterns and trends rather than individual cases. The classifier is a probabilistic model, not a definitive arbiter of misconduct. As such, all flagged papers represent statistical predictions based on textual features and should be interpreted as signals requiring human judgment and further verification, not as confirmed cases of fraud.

### Future directions and implications

Our model could be continuously improved by updating the training set with the latest confirmed paper mill papers. Because only titles and abstracts were used to train the model, incorporating full text data or selected sections of the full text has the potential to further enhance its performance. Future work could explore alternative, less computationally intensive training strategies, which might prove as effective as our fine tuned BERT classifier, considering that we evaluated only a limited set of configurations. Experimenting with other aggregation strategies and post hoc calibration methods may also help to improve the robustness and interpretability of model predictions.

We expect the paper mills to react and innovate as detection methods like ours threaten their income. The release of OpenAI’s ChatGPT-3.5 in 2022 and the rise of generative AI might further blur the boundaries between genuine and fabricated texts, rendering future automated detection of fraudulent features more challenging. While efforts to combat paper mills may evolve into an arms race,^1^ this problem has reached an unacceptable scale. Inaction risks allowing paper mills to spread further, potentially compromising entire journals and publishers—as already seen in the case of Hindawi.^5^

Our model is currently integrated into the online submission systems of three journals from a major publisher and is being used to screen cancer related manuscripts. To prevent unfair paper mill attributions, final decisions are always made by humans, using their expertise and a multitool detection approach to support decision making. The predictions are intended to be informative and to precede a second phase of scrutiny. Therefore, submissions are never rejected on the basis of the tool alone but only following serious integrity findings. Authors are not informed if their paper is flagged to prevent paper mills from adapting their templates. This collaboration did not influence the writing of this manuscript and no data were collected throughout this pilot testing phase.

### Conclusion

This study shows that using machine learning to identify papers resembling retracted paper mill papers from titles and abstracts is feasible and effective. Our findings reveal concerning trends in cancer research publishing. The rising percentage of flagged papers indicates that paper mills have grown in ambition and now target higher impact factor journals, highlighting the need for vigilance among journals, reviewers, and researchers. Although our model has clear limitations, it provides useful insights and highlights the need for collective awareness to curb the spread of paper mill publications.

**What is already known on this topic**

Paper mills are fraudulent organisations that produce manuscripts for sale using textual and structural templates, with an estimated prevalence of around 3% in biomedical research
Evidence suggests that cancer research may be a major target of paper mills, but the field lacks large scale estimates to assess the severity of the problem
Machine learning approaches have shown potential for detecting paper mill manuscripts from textual features, but no scalable approach has been publicly developed in cancer research

**What this study adds**

A scalable machine learning classifier can distinguish paper mill from genuine research using only titles and abstracts, suggesting that these sections contain template-like features
Large scale screening of cancer research showed that papers with textual similarities to paper mill manuscripts have increased over time and are widespread across the literature, including in high impact journals
Coordinated action from publishers and policy makers is urgently needed to address paper mill activity

## Data Availability

All data used in this study are publicly available: PubMed annual XML datasets (https://ftp.ncbi.nlm.nih.gov/pubmed/baseline/), Retraction Watch database,^27^ and the integrity experts’ dataset.^32^ The fine tuned models and code developed in this study are not publicly disclosed to prevent potential misuse by people seeking to evade fraud detection. The list of flagged PMIDs is available from the corresponding author (AGB) upon reasonable request.

## Web extra

Extra material supplied by authors

Web appendix: Supplementary files

## Notes

Contributors: AGB and JAB conceptualised the study. BS, AGB, DC, and JAB developed the methodology. BS developed and executed the code. BS, AGB, DC, and JAB performed the analysis. BS and AGB wrote the manuscript. BS, AGB, DC, and JAB reviewed and edited the manuscript. JAB and AGB secured the funding. AGB, JAB, and DC supervised the work. All work reported in the paper was performed solely by the authors. AGB is the guarantor of the study. The corresponding author attests that all listed authors meet authorship criteria and that no others meeting the criteria have been omitted.

Transparency: The corresponding author (AGB) affirms that the manuscript is an honest, accurate, and transparent account of the study being reported; that no important aspects of the study have been omitted; and that any discrepancies from the study as planned (and, if relevant, registered) have been explained.

Dissemination to participants and related patient and public communities: This study did not involve human participants. Findings will be shared with the scientific community through publication, media interviews, press coverage, social media, and presentations focused on research integrity.

Provenance and peer review: Not commissioned; externally peer reviewed.
