# Supplementary material for: Machine learning based screening of potential paper mill publications in cancer research: methodological and cross sectional study
Source: BMJ. 2026 Jan 29;392:e087581. doi: 10.1136/bmj-2025-087581 (PMC12853418; doi:10.1136/bmj-2025-087581)
Supplement: Supplementary file 1 — Web appendix: Supplementary files [file scab087581.ww.pdf]

Supplementary files

Supplementary File 1: Filtering strategy

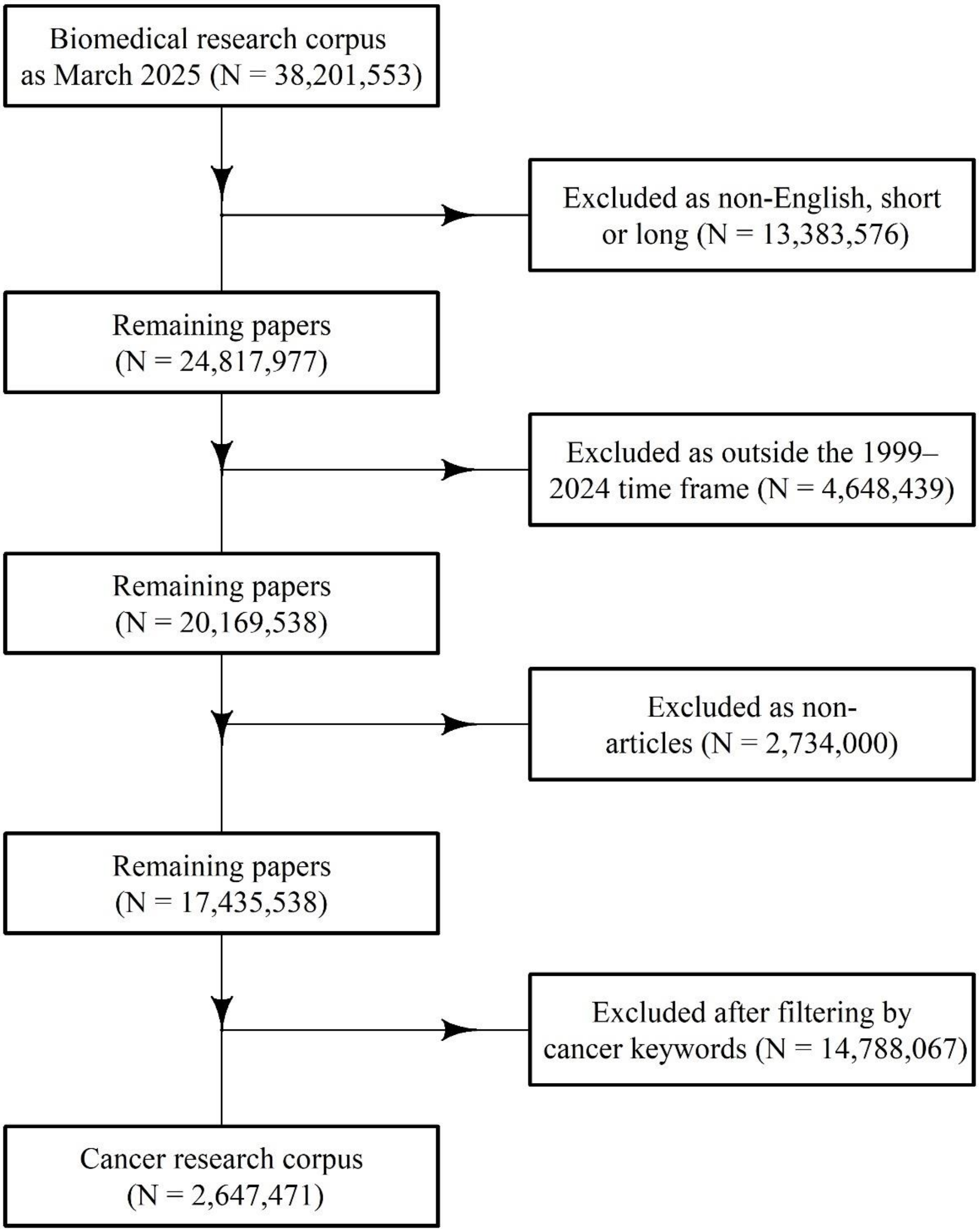

Figure S1: Flow diagram showing all preprocessing steps and exclusions applied to derive the final cancer research corpus. Cancer keywords are provided in Box 1 of the main manuscript.

Supplementary File 2: Supplementary methods

Affiliation-Based Country Indexing

The country of origin for each cancer research paper was inferred from the first author's affiliation. Affiliation addresses were processed to search for country names or common acronyms such as UK or UAE, using the ISO 3166-1 standard via the *pycountry* package[1]. For 432,145 papers, the country of origin could not be identified due to missing or incomplete affiliation information. In cases where the country name was missing but the rest of the affiliation was present, the addresses were passed through Google FLAN-T5-small[2] to infer the most likely country. Ultimately, 41,007 papers lacked any affiliation data, and for 89,446 others, the country could not be determined from incomplete affiliation information – resulting in a total of 130,453 papers labelled as 'No Data'. These data were excluded from country-related visualisations.

Publisher Identification

Using the journal ISSNs linked to papers from the cancer research corpus, the publishers were identified using the SCImago database[3]. A total of 137,361 (5%) papers could not be associated with a publisher via their ISSN, as the corresponding ISSNs were not indexed in SCImago.

Over the years 1999–2024, 1,822 publishers were identified as having contributed to the publication of the remaining 2,510,110 papers that included ISSNs. To simplify the analysis, only major publishers were retained in the dataset: publishers were ranked in descending order using the number of cancer-related publications, and those cumulatively accounting for 95% of cancer papers were retained. This selection process resulted in the retention of 212 out of 1,822 publishers, covering 2,384,049 papers (95%). The remaining 1,610 publishers each contributed to 126,061 papers over the years 1999–2024.

The 212 selected publishers were assigned to their parent companies. For example, *Taylor & Francis*, *Landes Bioscience*, and *Informa Healthcare* were grouped under *Informa*. Following this grouping, 136 distinct publishers were retained. Other publishers, as well as ISSNs for which no publisher could be identified, were assigned to the category 'Other', representing 263,422 papers (10%). These data were excluded from publisher-related visualisations.

Cancer-type identification

The type of cancer investigated in the papers was determined using AI labelling. Cancer type classification was based on cancer prevalence, adapted from the International Agency for Research on Cancer (IARC)[4] data (Box S2.1). Other cancer types were classified as 'unspecified cancer'. Data corresponding to 'unspecified cancer' were excluded from the visualisations.

Box S2.1: Cancer type classification.
1. breast cancer, 2. lung cancer, 3. colorectal cancer, 4. prostate cancer, 5. gastric cancer, 6. pancreatic cancer, 7. liver cancer, 8. ovarian cancer, 9. cervical cancer, 10. uterine cancer, 11. kidney cancer, 12. bladder cancer, 13. brain cancer, 14. skin cancer, 15. thyroid cancer,

16. blood cancer, 17. head and neck cancer, 18. oesophageal cancer, 19. bone cancer, and 20. unspecified cancer

Gastric cancer includes stomach and biliary tract cancers; brain cancer includes brain and central nervous system (CNS) cancers; blood cancer includes leukemia, lymphoma, and multiple myeloma; and head and neck cancer includes oral, laryngeal, pharyngeal, nasal, and salivary gland cancers.

A randomly selected subset of 15,000 papers from the cancer research corpus were pre-labelled using gpt-4o-mini from the OpenAI API on the title, abstract and MeSH terms of each paper. The prompt is displayed in Box S2.2 (Temperature = 0). A sample of 100 papers was double-annotated by a human reader (AB) and compared to GPT-4o-mini's annotations, resulting in an unweighted Cohen's Kappa of 0.81 (95% CI 0.72 to 0.89). All 15,000 labelled papers were used to train a BioBERT[5] model for a text-classification task to label the whole cancer research corpus (with accuracy = 0.95, F1-score micro = 0.94, F1-score macro = 0.92).

Box S2.2: Prompt for cancer type classification
""" You will be given a medical abstract or paragraph.

Your task is to classify the specific type of cancer being discussed. Choose only one label from the list below.

Read the text carefully and identify whether a clearly defined cancer type is mentioned based on biological, anatomical or clinical context.

- If the text explicitly mentions a cancer that matches one of the listed types, select that type.
- If multiple cancer types are mentioned select the one that is most central to the study, based on experimental models (cell lines, animal models), patient population or focus of the abstract.
- If the text refers to a cancer but the type is unclear, general, or not on the list, select unspecified cancer.

Respond with exactly one label, using the list below:

1. breast cancer
2. lung cancer
3. colorectal cancer #Includes colon and rectal cancer
4. prostate cancer
5. gastric cancer # Includes stomach and biliary tract cancers
6. pancreatic cancer
7. liver cancer
8. ovarian cancer
9. cervical cancer
10. uterine cancer
11.kidney cancer
12. bladder cancer
13. brain cancer # Includes brain and central nervous system (CNS) cancers
14. skin cancer
15. thyroid cancer
16. blood cancer # Includes leukemia, lymphoma and multiple myeloma

17. head and neck cancer #Include oral, laryngeal, pharyngeal, nasal and salivary gland cancers
18. oesophageal cancer
19. bone cancer
20. unspecified cancer

Now classify the following text:"""

Cancer research classification

As research objectives are often diverse and cannot always be captured by a single label, a multi-label classification approach was adopted to categorise the cancer research objectives using AI-based labelling. The classification scheme was built around a set of key research aims relevant to cancer research adapted from NCI – National Cancer Institute[6] research areas (see Box S2.3). Importantly, clinical trials were excluded from this classification, as such papers were filtered out of the cancer research corpus (due to our focus on preclinical research).

Box S2.3: Cancer research classification
1. Cancer biology and fundamental research, 2. Treatment development or evaluation, 3. Diagnosis and prognosis, 4. Prevention, 5. Survivorship, supportive care, and end-of-life, 6. Epidemiology and population studies, 7. Health systems, policy, and implementation

A representative subset of 12,000 papers was randomly sampled from the cancer research corpus and pre-labelled using the GPT-4o-mini model from the OpenAI API, based on each paper's title, abstract, and MeSH terms. The prompt is displayed in Box S2.4 (Temperature = 0). A sample of 100 papers was double-annotated by a human reader (AB) and compared to GPT-4o-mini's annotations. We computed Cohen's Kappa coefficients for each class: 1 = 0.74, 2 = 0.56, 3 = 0.57, 4 = 0.86, 5 = 0.65, 6 = 0.70, and 7 = 0.64. The macro-averaged Kappa (unweighted mean across classes) was 0.68, and the micro-averaged Kappa (weighted by class frequency) was 0.66. All 12,000 annotated papers were then used to fine-tune a BioBERT model for a multi-label classification task, enabling automated labelling of the entire corpus (with multi-label accuracy = 0.7, F1-score micro = 0.9, F1-score macro = 0.87).

Box S2.4: Prompt for cancer research classification
""" You will be given a medical abstract or paragraph.

Your task is to classify the main objectives of the cancer research study described in the text. Pick one or more categories from the list below that match the goals of the study, not the methods used, data collected or secondary details.

Respond with the list of codes that apply, separated by commas (BIO, THER).

Categories:

- BIO - Understanding cancer biology
- PREV - Prevention
- DIAG - Diagnosis and prognosis
- THER -Treatment development or evaluation
- IMPL - Health systems, policy, and implementation
- SURV - Survivorship, supportive care and end-of-life

- EPID - Epidemiology and population studies

Choose all codes that apply based on the primary objectives of the study.

Now classify the following text:"""

SCImago Journal Impact Factor retrieval

The SCImago Journal Impact Factor (SJIF) was retrieved for each journal from the SCImago database from 1999 to 2024. An SJIF value was available for 8,701 journals, while the remaining 2,931 journals were either not found or not indexed in SCImago. Each paper was matched with the SJIF corresponding to journal and year of publication. As a result, 2,469,177 papers (93%) were assigned an SJIF value, while 178,294 papers (7%) were labelled as 'no SJIF'.

Hyperparameter optimisation

The search space was pre-restricted through empirical testing, allowing the batch size to 32, as this setting yielded better performance. The number of training epochs was fixed at 10 to allow sufficient training time. The evaluation criterion was set to the evaluation loss to ensure greater model stability and prediction accuracy. Only the learning rate, weight decay, warmup ratio and scheduler type were optimised. Please note that this optimisation scheme only applies to the BERT model used for paper mill paper classification.

An initial hyperparameter search consisting of 100 trials was conducted using a guided exploratory strategy with random parameter combinations to identify promising configurations. The learning rate was searched within the range of 5e-6 to 5e-5, weight decay within 0.005 to 0.3, scheduler type between linear and cosine and the warmup ratio between 0% and 25% of the total training steps.

This was followed by a second, more focused adaptive search consisting of 50 trials to further refine the best-performing configuration. The top 10% of trials (based on evaluation loss) from the initial search were used to guide this step, with the goal of selecting the best-performing and most stable configuration. The learning rate search space was narrowed to the 1e-5 to 2e-5 range, and the warmup ratio to the 0.015 to 0.020 range. The weight decay showed an optimal range between 0.02 and 0.03. The scheduler type was fixed to cosine, with 'cosine with restarts' also evaluated. Final optimization results indicated the following parameters: a learning rate of 1.4e-5, a weight decay of 0.025, 15% warm-up steps, and a basic cosine scheduler type.

The final BERT model was trained for 2 hours using the optimised and fixed parameters and the 2100-training step checkpoint was selected (2 epochs), and its weights were merged with the initial model. After testing, the mean-over-probabilities method was preferred over logistic regression as the aggregation method, since the latter achieved similar performance (logistic regression: Accuracy = 0.91, Sensitivity = 0.92, and Specificity = 0.91). The decision threshold for flagging suspect papers was set at a probability of 0.6019, based on ROC curve optimisation. Large scale inferences on the cancer research corpus were made using a Tesla V100S-PCIE-32GB GPU over approximately 12 hours.

Supplementary references

1. Janssens, A. pycountry (version 23.12.11) [Python library]. https://pypi.org/project/pycountry/ (2023).
2. Won Chung, H. *et al.* Scaling Instruction-Finetuned Language Models. *J. Mach. Learn. Res.* **25**, 1–53 (2024).
3. SCImago. SJR — SCImago Journal & Country Rank. http://www.scimagojr.com.
4. International Agency for Research on Cancer (IARC). https://gco.iarc.who.int/en.
5. Lee, J. *et al.* BioBERT: A pre-trained biomedical language representation model for biomedical text mining. *Bioinformatics* **36**, 1234–1240 (2020).
6. National Cancer Institute (NCI). https://www.cancer.gov/.

Supplementary File 3: Cancer research corpus breakdown

Table S3: Summary of the cancer research corpus. The years have been displayed in descending chronological order and the number of rows has been set to the number of years (26). The other columns are displayed in descending order according to their counts.

Year	Count	Publisher	Count	Country	Count	Cancer type	Count	Research area	Count
2024	207,075	Elsevier	526,018	United States	622,867	breast cancer	280,597	BIO	1,629,283
2023	173,098	Springer Nature	407,097	China	497,672	blood cancer	211,006	THER	1,308,843
2022	194,774	John Wiley & Sons	283,946	Japan	161,240	lung cancer	190,293	DIAG	1,007,718
2021	194,902	MDPI	127,408	Italy	113,064	colorectal cancer	178,679	EPI	288,502
2020	171,656	Informa	116,511	Germany	107,407	liver cancer	136,719	PREV	171,272
2019	150,090	Wolters Kluwer	106,123	United Kingdom	99,679	brain cancer	136,285	IMPL	149,058
2018	139,756	Frontiers Media	73,020	South Korea	73,932	head and neck cancer	108,606	SURV	125,653
2017	133,885	Oxf. Univ. Press	66,374	India	71,546	prostate cancer	106,763		
2016	126,888	Am. Assoc. for Can. Res.	54,995	Canada	67,322	skin cancer	90,893		
2015	122,200	Spandidos Publications	49,796	France	63,608	gastric cancer	82,690		
2014	114,790	Am. Chemical Soc.	49,350	Spain	46,921	pancreatic cancer	58,684		
2013	105,424	PLoS	40,245	Australia	46,641	cervical cancer	58,163		
2012	97,137	SAGE Publications	35,423	Netherlands	44,242	ovarian cancer	51,782		
2011	84,207	Rapamycin Press LLC	24,018	Taiwan	40,951	kidney cancer	47,990		
2010	76,959	Roy. Soc. of Chem.	22,082	Iran	33,935	bone cancer	39,433		
2009	70,297	Bentham Science Publishers	21,868	Brazil	33,252	bladder cancer	33,549		
2008	65,188	BMJ Publ. Group	20,961	Turkey	30,346	thyroid cancer	32,477		
2007	60,935	Int. Inst. of Anticancer Res.	18,995	Poland	26,161	esophageal cancer	31,655		
2006	54,897	Karger Publishers	16,562	Sweden	25,829	uterine cancer	27,667		
2005	50,785	Mary Ann Liebert	15,742	Switzerland	20,378				
2004	47,336	Am. Soc. for Biochem. and Mol. Biol.	15,290	Denmark	17,177				
2003	44,609	AME Publ. Co.	13,033	Belgium	16,621				
2002	42,680	E-Cent. Publ. Corporation	10,510	Greece	15,347				
2001	40,916	IOS Press	10,302	Israel	15,092				
2000	39,441	Ivyspring Int. Publisher	10,069	Egypt	14,618				
1999	37,546	Nat. Academy of Sciences	9,639	Austria	13,930				

Supplementary File 4: Paper mill data

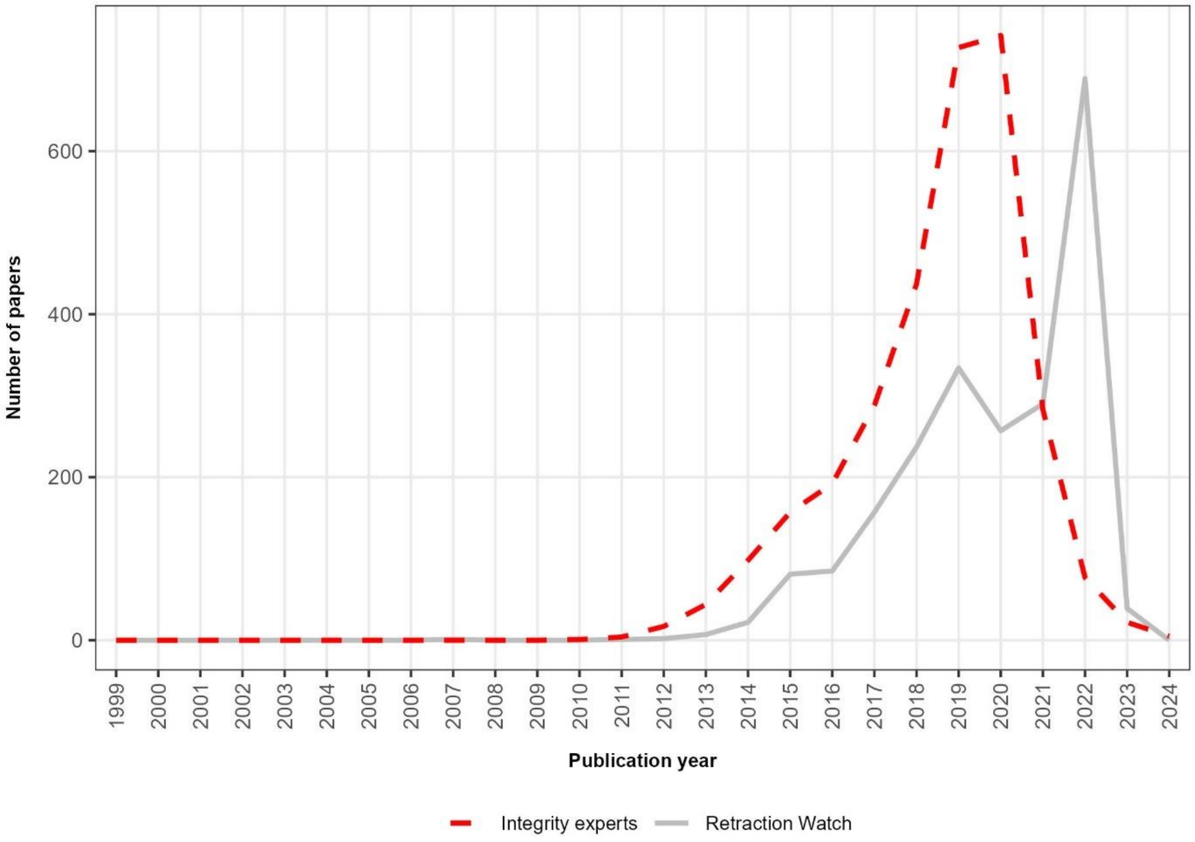

Figure S4: Distribution of retracted paper mill papers in the Retraction Watch (2,202 papers) and suspected paper mill papers from the integrity experts' set (3,094 papers). The Retraction Watch set is a solid grey line while the experts' set is a red dashed line. All papers present in both datasets (~1,000 papers) were removed from the integrity experts' set. The Retraction Watch dataset was used for training, validation, and testing, while the integrity experts' dataset was used exclusively for testing.

Table S4.1: Ten most frequent unigrams, bigrams, and trigrams found in the titles of retracted paper mill papers indexed by Retraction Watch.

Unigram	Count	Bigram	Count	Trigram	Count
cancer	1,019	cancer cells	195	cell lung cancer	127
cell	672	lung cancer	194	small cell lung	127
cells	473	migration invasion	173	non small cell	125
mir	441	cell proliferation	145	long noncoding rna	123
microrna	439	cell lung	134	non coding rna	86
targeting	398	signaling pathway	131	long non coding	84
proliferation	382	long noncoding	130	proliferation migration invasion	79
carcinoma	348	breast cancer	128	squamous cell carcinoma	59
invasion	338	small cell	127	epithelial mesenchymal transition	57
inhibits	335	non small	126	cell proliferation invasion	43

Table S4.2: Five most frequent publishers, first author country of affiliation, cancer types investigated and main research axes among retracted paper mill papers indexed by Retraction Watch. Entries with no country data (n = 34) and unspecified cancer types (n = 229) were excluded from the table. Research area labels: BIO - Cancer biology and fundamental research; THER - Treatment development or evaluation; DIAG - Diagnosis and prognosis; SURV - Survivorship, supportive care, and end-of-life; and PREV - Cancer prevention.

Publisher	Count	Country	Count	Cancer type	Count	Research area	Count
John Wiley & Sons	1,124	China	2,010	lung cancer	293	BIO	1,790
Spandidos Publications	340	India	51	liver cancer	180	THER	1,375
Informa	160	Saudi Arabia	25	colorectal cancer	167	DIAG	639
Verduci Editore s.r.l.	153	Iran	19	brain cancer	160	SURV	71
Springer Nature	69	Pakistan	8	gastric cancer	145	PREV	48

Table S4.3: Ten most frequent unigrams, bigrams, and trigrams found in the titles of suspected paper mill papers listed in the integrity experts' dataset.

Unigram	Count	Bigram	Count	Trigram	Count
cancer	1,642	cancer cells	423	non coding rna	231
cell	1,227	signaling pathway	306	long non coding	226
cells	953	migration invasion	280	non small cell	159
mir	783	cell proliferation	266	small cell lung	159
microrna	732	lung cancer	250	cell lung cancer	155
proliferation	723	coding rna	236	epithelial mesenchymal transition	148
promotes	671	non coding	231	long noncoding rna	145
pathway	619	long non	226	proliferation migration invasion	145
invasion	612	hepatocellular carcinoma	221	cell cycle arrest	116
carcinoma	579	breast cancer	197	squamous cell carcinoma	111

Table S4.4: Five most frequent publishers, first authors country of affiliation, cancer types investigated and main research areas among suspected paper mill papers listed in the integrity experts' dataset. Research area labels: BIO - Cancer biology and fundamental research; THER - Treatment development or evaluation; DIAG - Diagnosis and prognosis; EPID - epidemiology and population studies; and PREV - Cancer prevention.

Publisher	Count	Country	Count	Cancer type	Count	Research area	Count
Springer Nature	433	China	2,927	lung cancer	357	BIO	3,033
John Wiley & Sons	389	United States	5	liver cancer	295	THER	1,973
Spandidos Publications	300	Japan	2	colorectal cancer	267	DIAG	708
Elsevier	291	Taiwan	2	gastric cancer	244	EPID	81
Informa	264	Ukraine	1	brain cancer	223	PREV	13

Supplementary File 5: Model pre-assessment

We have conducted preliminary experiments with BERT and other BERT-based models, including RoBERTa, BioBERT, PubMedBERT, Longformer, and Clinical Longformer. These alternatives were selected to assess whether biomedical-specific pretraining or extended input capacity (up to 4,096 tokens, compared to BERT's 512-token input limit) could enhance classification performance. While PubMedBERT and BioBERT slightly outperformed BERT in our empirical experiments, we did not consider these differences as important given BERT's already high performance (*Table S5*). As neither domain-specific pretraining nor the ability to process longer text sequences provided a clear advantage over BERT and given the potential risk of data leakage in domain-specific models, we chose to retain BERT.

BERT, RoBERTa, BioBERT, PubMedBERT, Longformer, and Clinical Longformer were not trained and assessed on the same data as those used in the manuscript. Although the overall methodology was identical for the origin of cases and the selection of controls, early testing was conducted in June 2024. Approximately 1,400 paper mill papers were used for training, while the external validation set comprised the remaining 200 Retraction Watch papers and 2,000 papers identified by Integrity Experts. Paper mill papers were matched with an equal number of controls (n = 3,400), selected from Chinese (5%), Taiwanese (28%), Finnish (11%), Swedish (11%), and Norwegian papers (11%), as well as papers published in high-impact journals (33%). Accuracy, sensitivity, and specificity were derived from the confusion matrix using a probability threshold to prioritise specificity and minimise false positives.

Table S5: Initial empirical comparison of six models. Preliminary experiments compared BERT, RoBERTa, BioBERT, PubMedBERT, Longformer, and Clinical Longformer to assess the impact of biomedical-specific pretraining and extended input capacity on classification performance, using Retraction Watch and integrity experts' data as June 2024. All metrics show similar predictive performance.

Model	Accuracy	Sensitivity	Specificity
BERT	0.96	0.98	0.95
RoBERTa	0.95	0.99	0.92
PubMedBERT	0.97	0.99	0.95
BioBERT	0.97	0.98	0.96
Longformer	0.96	0.96	0.96
Clinical Longformer	0.96	0.98	0.94

Supplementary File 6: Study diagram

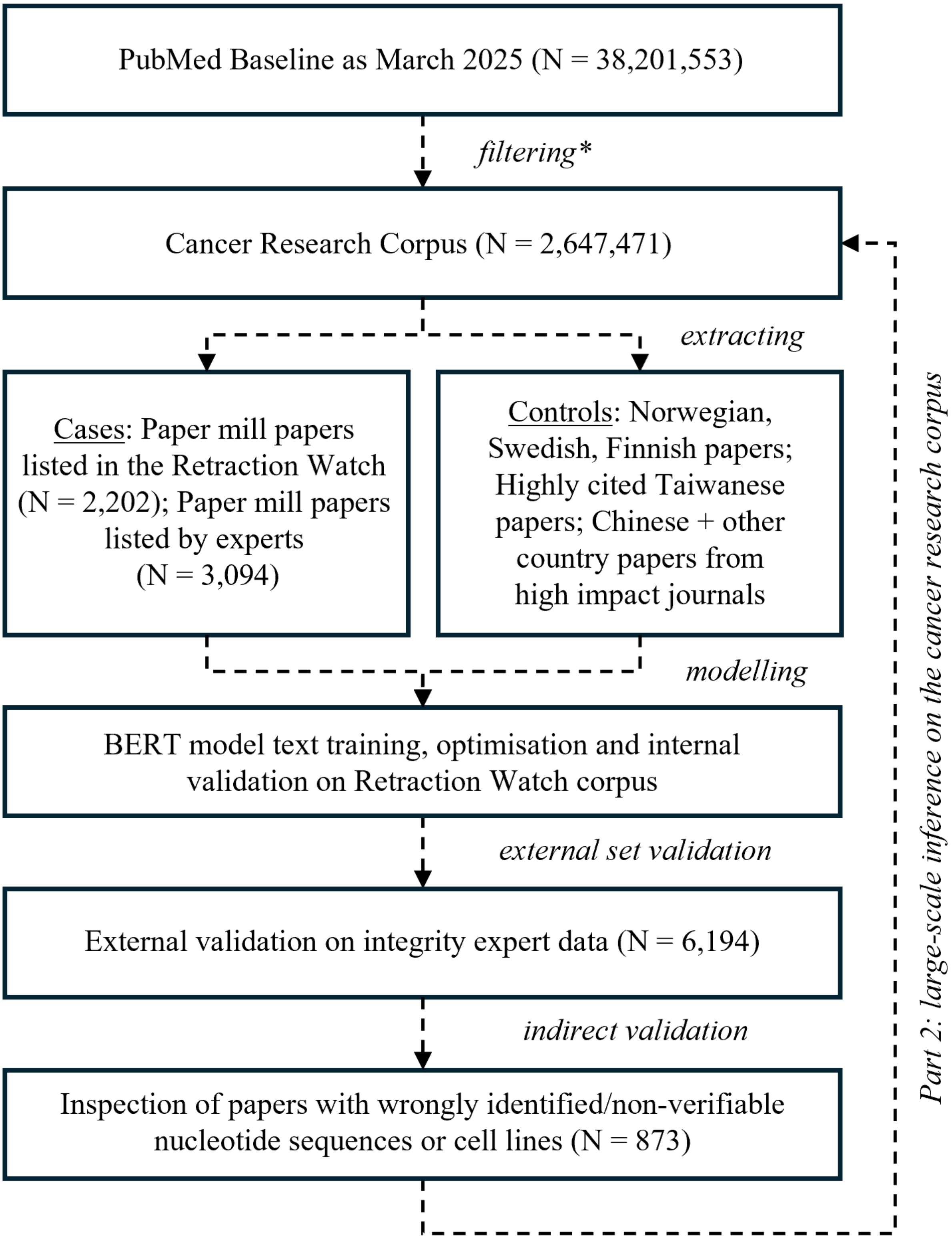

Figure S6: Study design and workflow for large-scale detection of papers textually similar to paper mill papers in the cancer research corpus. Filtering method (*) is shown in Figure S1.

Supplementary File 7: Examples of papers flagged with high probability

All three papers presented in Supplementary File 7 are retracted papers tagged as '*Paper Mill*' in the Retraction Watch database. They were selected from the test set and were not seen by the model during training.

Retracted paper A

Model-predicted paper mill origin probability: **0.99**

Title: Long non-coding RNA SNHG14 contributes to gastric cancer development through targeting miR-145/SOX9 axis

Abstract: This study aimed to elucidate the roles of long non-coding RNA SNHG14 in gastric cancer development. LncRNA SNHG14 was markedly up-regulated in gastric cancer tissues and cells. Knockdown of SNHG14 significantly inhibited SGC-7901 cell viability, migration, invasion, and promoted cell apoptosis. In addition, miR-145 was negatively regulated by SNHG14 and the effects of SNHG14 knockdown on cell viability, apoptosis, migration, invasion, and the expression of apoptosis-related proteins and EMT-markers were reversed by inhibition of miR-145 at the same time. Furthermore, SOX9 was verified as a functional target of miR-145, and miR-145 regulated tumor malignant behaviors through regulating SOX9. Besides, knockdown of SNHG14 inhibited the expression of p-PI3 K, p-AKT, and p-mTOR and promoted PTEN expression, where miR-145 inhibition had opposite effects. Moreover, the activated PI3 K/AKT/mTOR pathway caused by miR-145 inhibition was counteracted after knockdown of SOX9. Our findings indicate that up-regulation of lncRNA SNHG14 may contribute to gastric cancer development via targeting miR-145/SOX9 axis and involving in PI3 K/AKT/mTOR pathway. SNHG14-miR-145/SOX9 axis may be a promising therapeutic strategy for gastric cancer treatment.

Retracted paper B

Model-predicted paper mill origin probability: **0.98**

Title: miR-451a Inhibits the Growth and Invasion of Osteosarcoma via Targeting TRIM66.

Abstract: The importance of microRNAs in regulating osteosarcoma development has been studied in recent years. However, the function of microRNA-451a in osteosarcoma growth is rarely investigated. Here, we explored the expression of microRNA-451a in osteosarcoma cell lines. Bioinformatic software, luciferase activity reporter assay, and Western blot were conducted to determine the association between microRNA-451a and tripartite motif-containing 66. Cell Counting Kit-8 assay and transwell assay were used to explore the regulatory effects of microRNA-451a on osteosarcoma cells. Moreover, we explored whether microRNA-451a modulates osteosarcoma cell biological activity by regulating tripartite motif-containing 66. The expression of microRNA-451a was found to be downregulated in osteosarcoma and negatively regulated the expression of tripartite motif-containing 66. Tripartite motif-containing 66 was further validated as a target of microRNA-451a. MicroRNA-451a inhibits the growth and invasion of osteosarcoma cell lines through targeting tripartite motif-containing 66. The miR-451a targets tripartite motif-containing 66 may provide novel therapeutic targets for the treatment of osteosarcoma.

Retracted paper C

Model-predicted paper mill origin probability: **0.98**

Title: SLCO4A1-AS1 mediates pancreatic cancer development via miR-4673/KIF21B axis.

Abstract: In this study, we intended to figure out the biological significance of long non-coding RNAs (lncRNAs) solute carrier organic anion transporter family member 4A1 antisense RNA 1 (SLCO4A1-AS1) in pancreatic cancer (PC). Cell counting kit-8, colony formation, wound healing, transwell, and flow cytometry experiments were performed to reveal how SLCO4A1-AS1 influences PC cell proliferation, migration, invasion, and apoptosis. Thereafter, bioinformatics analysis, RNA immunoprecipitation assay, luciferase reporter assay, and RNA pull-down assay were applied for determining the binding sites and binding capacities between SLCO4A1-AS1 and miR-4673 or kinesin family member 21B (KIF21B) and miR-4673. The results depicted that SLCO4A1-AS1 was upregulated in PC, and SLCO4A1-AS1 knockdown suppressed PC cell growth, migration, invasion, and induced cell apoptosis. Furthermore, SLCO4A1-AS1 was verified to modulate the expression of KIF21B by binding with miR-4673. SLCO4A1-AS1 exerted an oncogenic function in PC. The overexpression of SLCO4A1-AS1 aggravated the malignant behaviors of PC via the upregulation of KIF21B by sponging miR-4673. Our findings revealed a novel molecular mechanism mediated by SLCO4A1-AS1, which might play a significant role in modulating the biological processes of PC.

Supplementary File 8: Supplementary analysis of model misclassifications

Table S8. Characteristics of false negatives (n = 433) by research area, and title n-grams (unigrams, bigrams and trigrams). A Pearson's chi-squared test of independence was carried out for each category of the multi-label variables (multiple testing). P-values were corrected with the Benjamini-Hochberg method. The percentage is shown both within the false negatives and in the overall pooled validation dataset (n = 6,745). The difference represents false negatives minus overall. Research area codes: BIO – Cancer biology and fundamental research; PREV – Prevention; DIAG – Diagnosis and prognosis; THER – treatment development or evaluation; IMPL – Health systems, policy, and implementation; SURV – Survivorship, supportive care, and end-of-life; and EPID – epidemiology and population studies.

Variables	Percent in False Negatives (%)	Percent in Overall (%)	Diff (%)	χ^2 p-value
Research area:				
BIO	86%	87%	−1%	0.51
PREV	2%	2%	0%	0.61
DIAG	25%	24%	1%	0.51
THER	54%	49%	5%	0.09
IMPL	0%	2%	−2%	0.03
SURV	1%	3%	−2%	0.11
EPID	18%	7%	11%	< 0.001
Unigrams:				
cancer	57%	40%	17%	< 0.001
cells	28%	20%	8%	< 0.001
cell	26%	23%	3%	0.29
mir	4%	12%	−8%	< 0.001
proliferation	9%	12%	−3%	0.04
Bigrams:				
cancer cells	13%	8%	5%	< 0.001
lung cancer	8%	5%	3%	< 0.001
breast cancer	9%	6%	3%	< 0.001
signaling pathway	6%	5%	1%	0.2
migration invasion	4%	5%	−1%	0.65
Trigrams:				
non coding rna	1%	4%	−3%	0.03
long non coding	1%	4%	−3%	0.03
small cell lung	3%	3%	0%	1
has non small cell	3%	3%	0%	0.88
has cell lung cancer	3%	3%	0%	0.46
